# Supplementary material for: Replicating viral vector platform exploits alarmin signals for potent CD8+ T cell-mediated tumour immunotherapy
Source: Nat Commun. 2017 May 26;8:15327. doi: 10.1038/ncomms15327 (PMC5458557; doi:10.1038/ncomms15327)
Supplement: Supplementary Information — Supplementary Figures and Supplementary References [file ncomms15327-s1.pdf]

Supplementary Figure 1

a Gating for artLCMV- or r3LCMV-infected BHK-NP cells

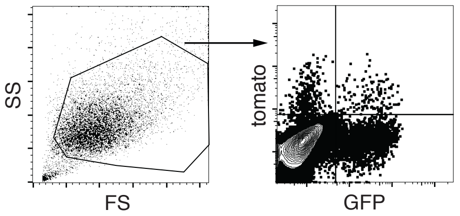

b Gating for MHC class I tetramer-binding CD8+ T cells

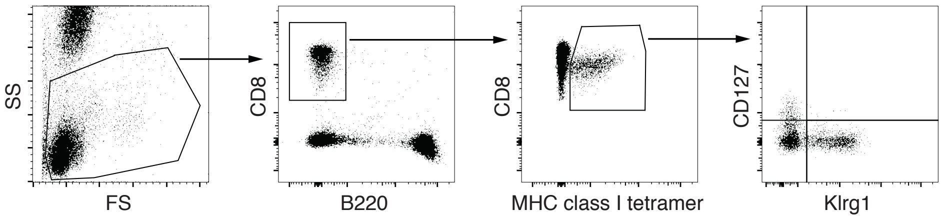

c Gating for cytokine-producing CD8+ T cells

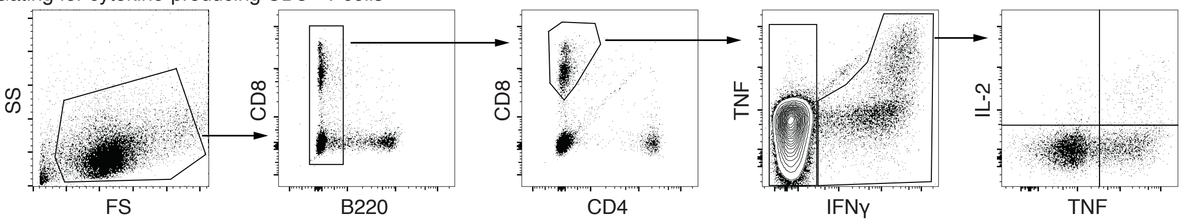

d Gating for dendritic cells and macrophages

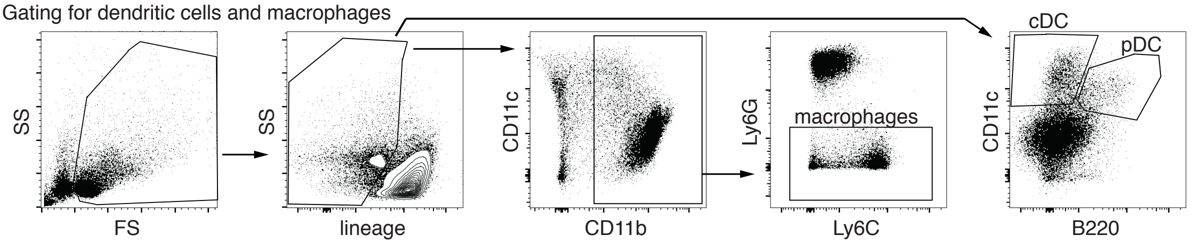

e Gating for virus-infected IL-33-reporting stromal cells in spleen

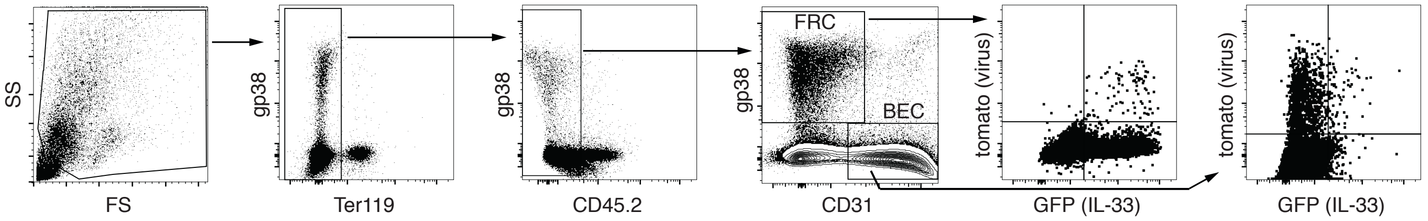

f Gating for tumor cells and tumor-infiltrating CTL

g

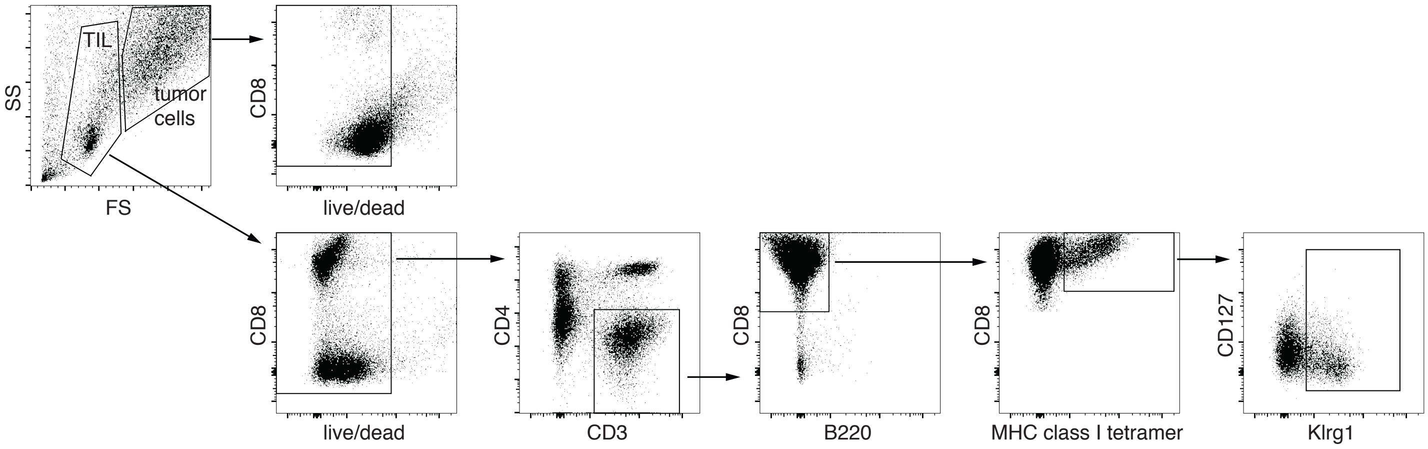

h Gating for peripheral blood monocytes

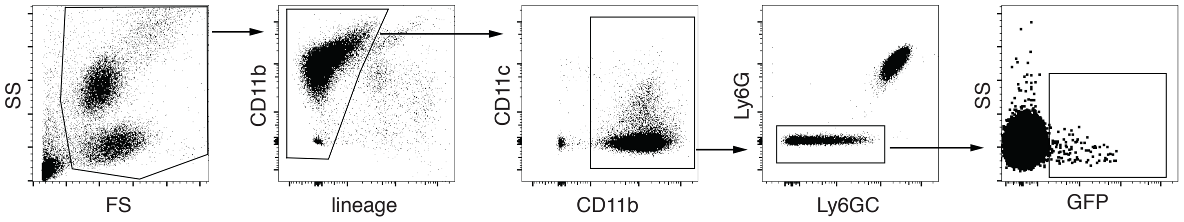

Supplementary Figure 1: Gating strategies for flow cytometric analyses

Gating strategies for flowcytometric analysis of LCMV-infected BHK-NP cells (**a**; data displayed in Fig. 1e), MHC class I tetramer binding $CD8^+$ T cells (**b**; data displayed in Figs. 4a-d, 4i, 4k, 5b-c, 7a, Supplementary Fig. 3b-c, Supplementary Fig. 5c-d), cytokine-producing $CD8^+$ T cells (**c**; data displayed in Figs. 4e-f, 4h), dendritic cells and macrophages (**d**; data displayed in Fig. 5a), splenic stromal cells (**e**; data displayed in Fig. 5d, Supplementary Fig. 4c-d), tumor cells (**f**; data displayed in Fig. 7b, Supplementary Fig. 5e), tumor-infiltrating CTL (**g**; data displayed in Fig. 7b, Supplementary Fig. 5e) and peripheral blood monocytes (**h**; data displayed in Supplementary Fig. 2c). Pre-gating for singlets and non-autofluorescent cells not displayed.

Supplementary Figure 2

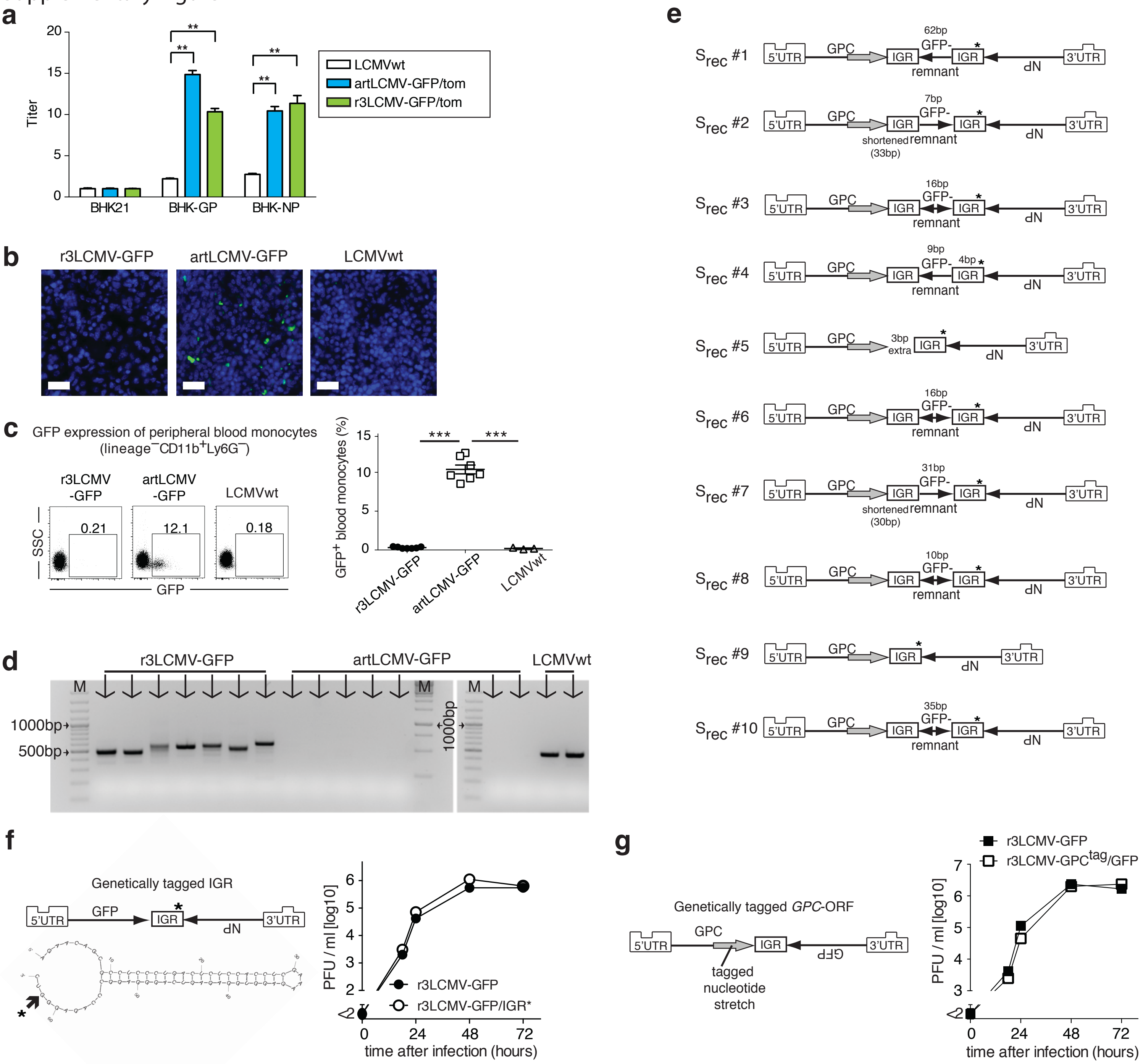

5' UTR / 3' UTR: 5' / 3' untranslated regions
IGR: intergenic region
S_{rec}: S segments derived from recombination of S_{GP} and S_{NP} segments

Supplementary Figure 2: artLCMV attenuation and long-term transgene expression as opposed to r3LCMV recombination and transgene loss

(**a**) LCMVwt, artLCMV-GFP/tom and r3LCMV-GFP/tom (grown on BHK-21 cells) were titrated on non-complementing BHK-21 cells, on GP-expressing BHK-GP cells and on NP-expressing BHK-NP cells. Titers on BHK-21 and BHK-GP cells were determined by NP-IFF, titers on BHK-NP by GP-IFF. The infectious titers obtained are expressed as a multiple of the titer of each respective virus as determined on BHK-21 cells (BHK-21 titers arbitrarily set to 1). Bars represent the mean+SEM of six replicates per group. *N* = 3. (**b**-**e**) We infected AGRAG mice with r3LCMV-GFP, artLCMV-GFP or LCMVwt (same experiment as in Fig. 2a-d) and analyzed them on d150. (**b**) Representative spleen sections were analyzed for GFP^+ cells on histological sections. n=3 (LCMVwt) or n=7 (r3LCMV-GFP, artLCMV-GFP). Scale bar 50 μm. (**c**) FACS analysis of peripheral blood monocytes ($lineage^-CD11b^+Ly6G^-$). Representative FACS plots are shown. Symbols represent individual mice, bars represent the mean±SEM of n=3 (LCMVwt) or n=7 (r3LCMV-GFP, artLCMV-GFP) mice per group. (**d**,**e**) We performed RT-PCR analysis of viral RNA in serum using primers binding to *NP* and *GPC* sequences, thus detecting RNA molecules containing both viral genes. (**d**) DNA electrophoresis of PCR products. Each lane corresponds to an individual mouse. M: DNA size marker. *N* = 2. (**e**) Amplicons in (**d**) were subject to Sanger sequencing. A schematic shows deducted S_{rec} segments from 10 animals, housed in small groups in a total 4 cages in two independent series of experiments to limit virus transmission between animals. (**f**) For genetic tracing of the IGR sequences in S_{rec} segments, originating from the *GPC*- and *NP*-expressing S segments of r3LCMV-GFP, respectively, we introduced a genetic tag into the IGR of the $S_{GP/GFP}$ segment (IGR*; arrow in the stem loop structure pointing to the deleted residue at position 1,637 of Genbank accession number DQ361065.2). Viruses with and without genetic tag in the IGR (r3LCMV-GFP/IGR*; r3LCMV-GFP) grew identically on BHK-21 cells. Symbols represent the mean±SEM of three replicates per group (hidden in the symbol size). *N* = 2. (**g**) To exclude laboratory contaminations, plasmid DNA, RNA or virus, as potential sources

of S_{rec} amplicons in the experiments of (**d**), we used r3LCMV viruses containing a *GPC* with its 255 C-terminal nucleotides originating from a codon-optimized sequence ("GPC^{tag}"). A combination of GPC^{tag} with *NP* on a single RNA or DNA molecule did not exist, neither in our laboratory nor in nature, and hence such RT-PCR products were unequivocal evidence of viral RNA recombination. A r3LCMV virus harboring GPC^{tag} (r3LCMV-GPC^{tag}/GFP) and a control virus with a wt *GPC* sequence (r3LCMV-GFP) showed identical growth on BHK-21 cells. Symbols represent the mean±SEM of three replicates per group (error bars hidden in the symbol size). *N* = 2. Data in (**a**) and (**c**) were analyzed by one-way ANOVA with Bonferroni post-test. $^{**}P<0.01$ and $^{***}P<0.001$.

Supplementary Figure 3

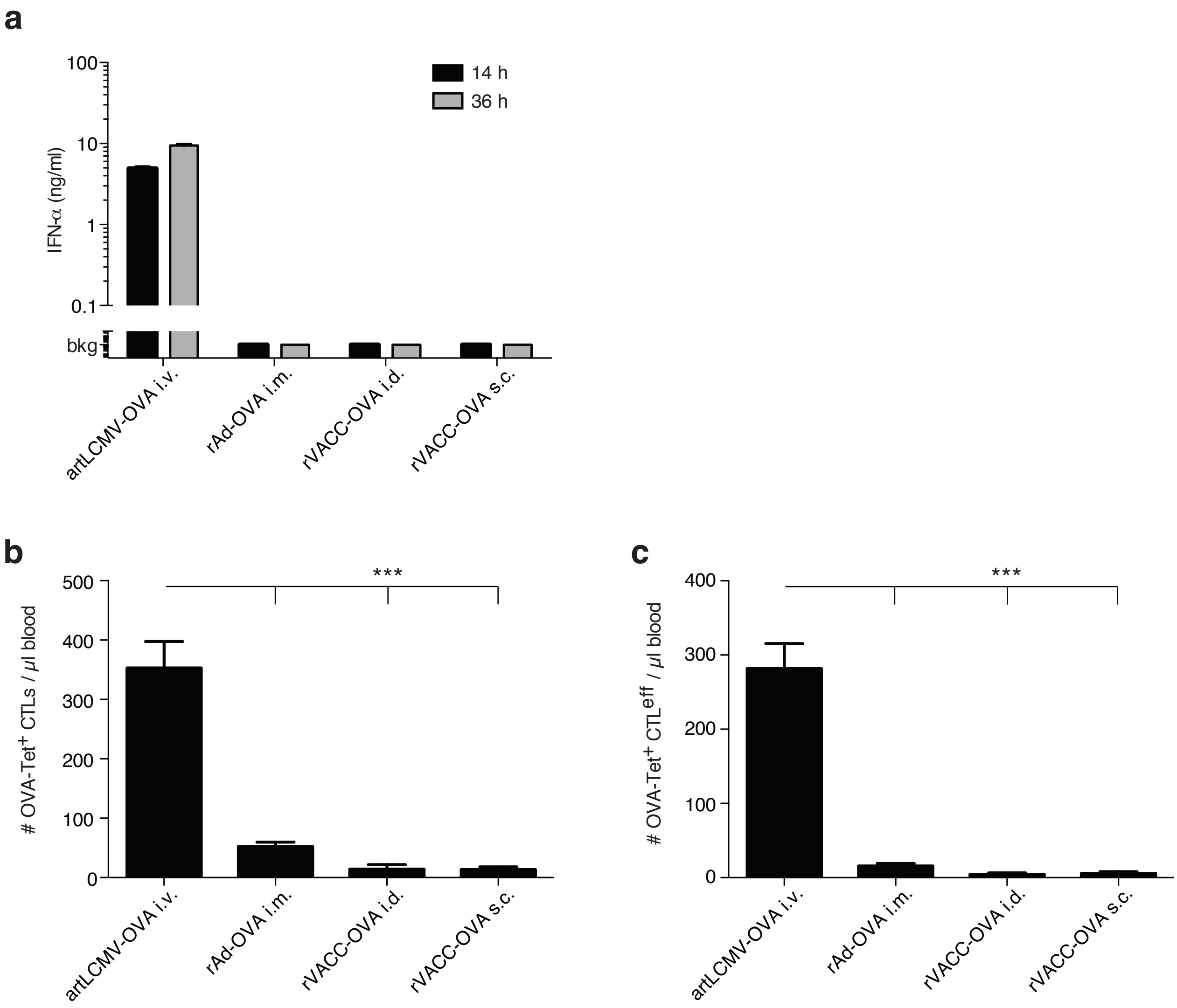

Supplementary Figure 3

We immunized mice with artLCMV-OVA i.v., rAd-OVA i.m., rVACC-OVA s.c. or rVACC-OVA i.d. and determined serum IFN-α levels at the indicated time points (**a**). Bars represent the mean+SEM of 5 mice. We enumerated OVA-specific CTLs (**b**) and CTL^{eff} ($Klrg1^{+}CD127^{-}$, **c**) in blood on day 8. Bars represent the mean+SEM of 5 mice. Data in (**b**) and (**c**) were analyzed by one-way ANOVA with Bonferroni post-test. ***P<0.001.

Supplementary Figure 4

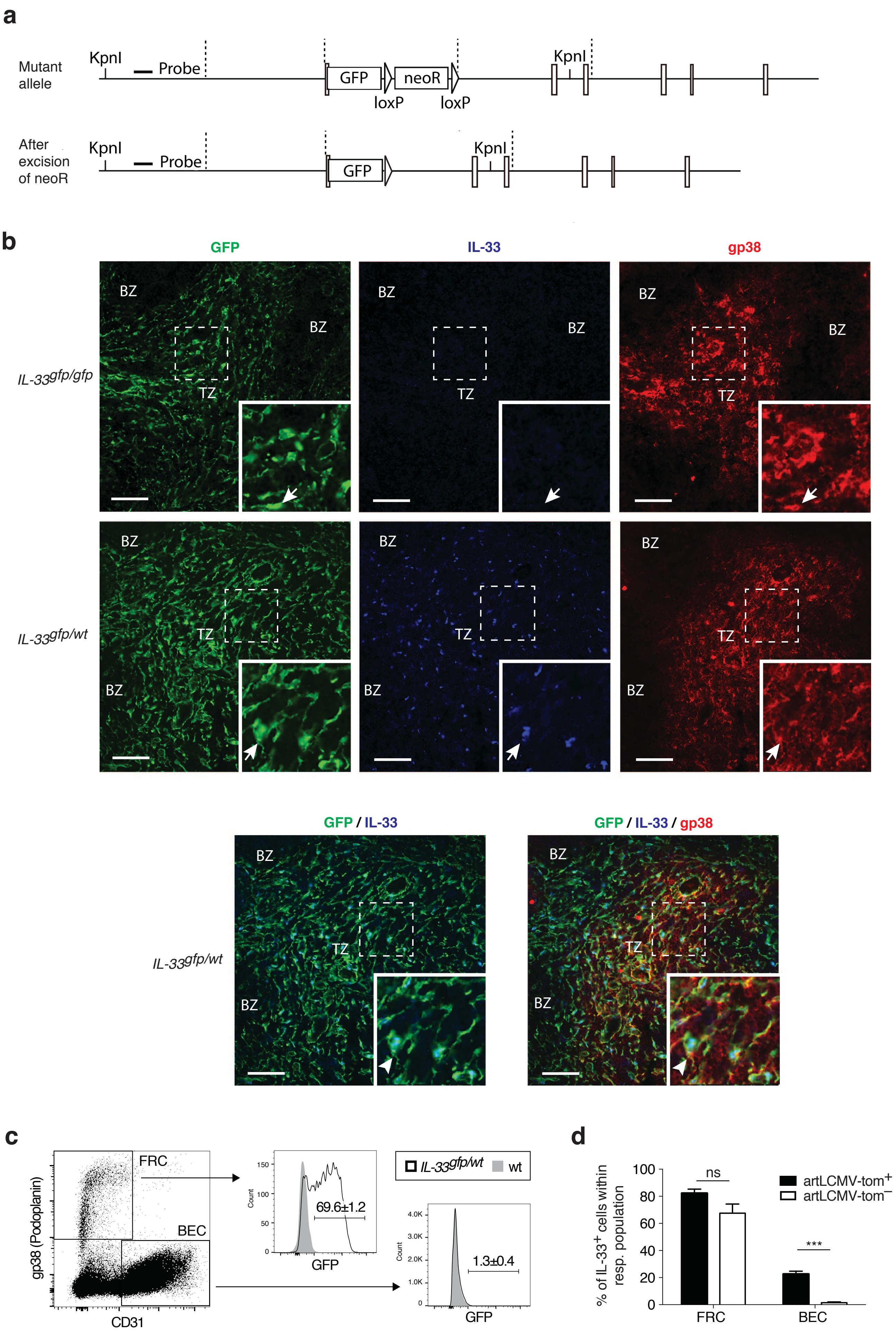

Supplementary Figure 4: Generation and validation of an IL-33 reporter mouse and characterization of its lymphoid stromal cell compartment.

(**a**) Targeted IL-33 locus[1] prior to and after Cre-lox recombination to remove the neomycin cassette (which putatively exerts an antisense effect on GFP expression), thus unleashing GFP reporting from the targeted locus. (**b**) Immunostainings of naïve spleen sections from *IL-33*$^{gfp/gfp}$ and *IL-33*$^{gfp/wt}$ mice: Representative single stains are shown for the indicated markers, together with merges of two or three of them. Magnifications of selected T zone regions, as indicated by a dashed-line square, are shown as inserts. Exposure and image processing were identical for the two types of mice. Note that in spleens of *IL-33*$^{gfp/wt}$ mice, nuclear IL-33 protein co-localizes with IL-33 promoter-driven cytoplasmic GFP. In contrast, *IL-33*$^{gfp/gfp}$ mice are IL-33 deficient and show only GFP but no IL-33 protein expression. Most of the cells expressing both GFP and IL-33 protein are localized within splenic T zones of *IL-33*$^{gfp/wt}$ mice and represent T zone FRCs, as characterized by gp38 (podoplanin) expression and reticular morphology. BZ (B cell-rich zone or follicle) and TZ (T cell-rich zone or periarteriolar sheeth) represent the white pulp of the spleen. Scale bars: 50 μm. (**c**) FACS analysis of spleen stroma (CD45.2$^-$Ter119$^-$ cells) from hemizygous IL-33 reporter mice (*IL-33*$^{gfp/wt}$) in comparison to C57BL/6 wt mice (wt). FRCs (gp38$^+$CD31$^-$) and BECs (gp38$^-$CD31$^+$) were identified and IL-33-reporting (GFP$^+$) cells are shown in histograms. Values show the mean±SEM of three mice per group. *N* = 3. (**d**) FACS analysis of spleen stroma (CD45.2$^-$Ter119$^-$ cells) from infected hemizygous IL-33 reporter mice (*IL-33*$^{gfp/wt}$) four days after infection with artLCMV-tom. Quantifications of IL-33-reporting (GFP$^+$) cells within the infected (tom$^+$) and uninfected (tom$^-$) FRC (gp38$^+$CD31$^-$) and BEC (gp38$^-$CD31$^+$) populations are shown. Bars represent the mean+SEM of four mice per group. *N* = 3. Data in (**d**) were analyzed by unpaired two-tailed Student's *t* test. ns, not significant; ***P<0.001

Supplementary Figure 5

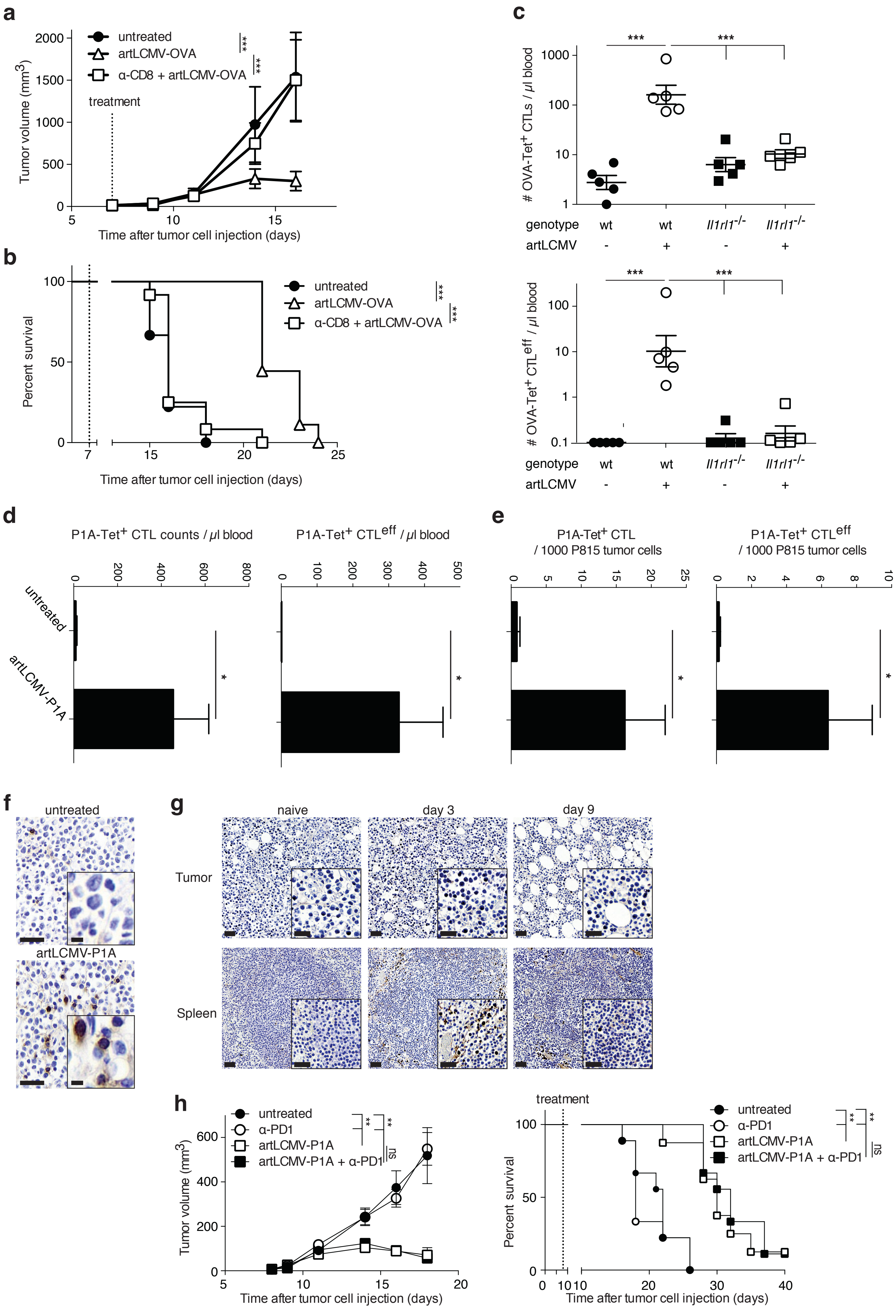

Supplementary Figure 5: IL-33 dependent CTL and CTL^{eff} responses in artLCMV-treated tumor-bearing mice and lack of detectable artLCMV antigen in tumors.

(**a**,**b**) We implanted EG7-OVA tumor cells subcutaneously into the flank of C57BL/6 mice. On day 7, when tumors became palpable, we treated them with artLCMV-OVA or left them untreated. CD8$^+$ T cell blocking antibody was administered on day 4, 6 and 11 after tumor cell injection. Tumor growth over time (**a,** terminated when the first animal was lost from follow-up owing to humane endpoint) and survival curves (**b**) are shown. Symbols represent the mean±SEM of nine mice (untreated, artLCMV-OVA) or twelve mice per group (α-CD8 + artLCMV-OVA). (**c**) We implanted EG7-OVA tumor cells subcutaneously into the flank of C57BL/6 (wt) and ST2-deficient (*Il1rl1*$^{-/-}$) mice. When tumor masses became palpable on d7, we treated the mice with artLCMV-OVA or left them untreated. Peripheral blood was analyzed nine days later (d16) by flow cytometry. We quantified total OVA-specific CTLs (upper graph) and effector CTLs (CTL^{eff}; Klrg1$^+$CD127$^-$, lower graph). Symbols represent individual mice, bars represent the mean±SEM. (**d**-**f**) We implanted P815 tumor cells subcutaneously into the flank of DBA/2 mice. When tumor masses became palpable on d9, we treated them with artLCMV-P1A or left them untreated. Peripheral blood (**d**) was analyzed 8 days later (d17), tumor tissue (**e**,**f**) on d18. We performed flow cytometry to enumerate the total number of P1A-tetramer-binding CTLs and CTL^{eff} (Klrg1$^+$) in blood and tumor as indicated (**d**,**e**). CTLs were gated as live CD8$^+$CD4$^-$CD3$^+$B220$^-$ lymphocytes in tumors, or as CD8$^+$B220$^-$ lymphocytes in peripheral blood. For normalization to P815 tumor cells, the latter were differentiated from infiltrating inflammatory cells by size and granularity. Symbols represent the mean+SEM of 5 (artLCMV-OVA) or 4 mice (all other groups) per group. Immunohistochemistry of tumors was performed 9 days after treatment (d18) to detect infiltrating CD8$^+$ T cells (**f**). Representative pictures from one out of 5 (artLCMV-OVA) or 4 mice (all other groups) per group are shown. Scale bars 10 μm (inset) and 50 μm (overview). (**g**) We implanted EG7-OVA tumor cells subcutaneously into the flank of C57BL/6 mice. On day 7, when tumors became palpable, we treated the mice with artLCMV-OVA or left them untreated. Three and nine

days later, tumor and spleen tissue was processed for detection of LCMV-infected (NP^+) cells by immunohistochemistry, showing viral infection in spleen but not detectably in the tumor. Representative pictures from one out of three to four animals are shown. Scale bars 50 μm inset and 100 μm overview. (**h**) We implanted P815 tumor cells subcutaneously into the flank of DBA/2 mice. When tumor masses became palpable on d9, we treated them with artLCMV-P1A, 12.5 mg/kg anti-PD1 antibody on d15 (earliest possible onset of the artLCMV-P1A-induced CTL response), d18, d22 and d25, a combination of artLCMV-P1A and anti-PD1 antibody or left them untreated. Tumor volumes (mean±SEM) and Kaplan-Meier survival curves of 8 (artLCMV-P1A) or 9 mice per group (all other groups) are shown (same data set as in Fig. 6 i,j). Tumor growth curves in (**a**) and (**h**) were analyzed by comparing the Area Under the Curve (AUC) by one-way ANOVA with Bonferroni post-test, survival data in (**b**) and (**h**) were analyzed by log-rank tests with Bonferroni correction. Data in (**c**) were analyzed by one-way ANOVA with Bonferroni post-test and data in (**d**) and (**e**) were analyzed by unpaired two-tailed Student's *t* test. ns, not significant; *$P<0.05$, **$P<0.01$ and ***$P<0.001$.

Supplementary Figure 6

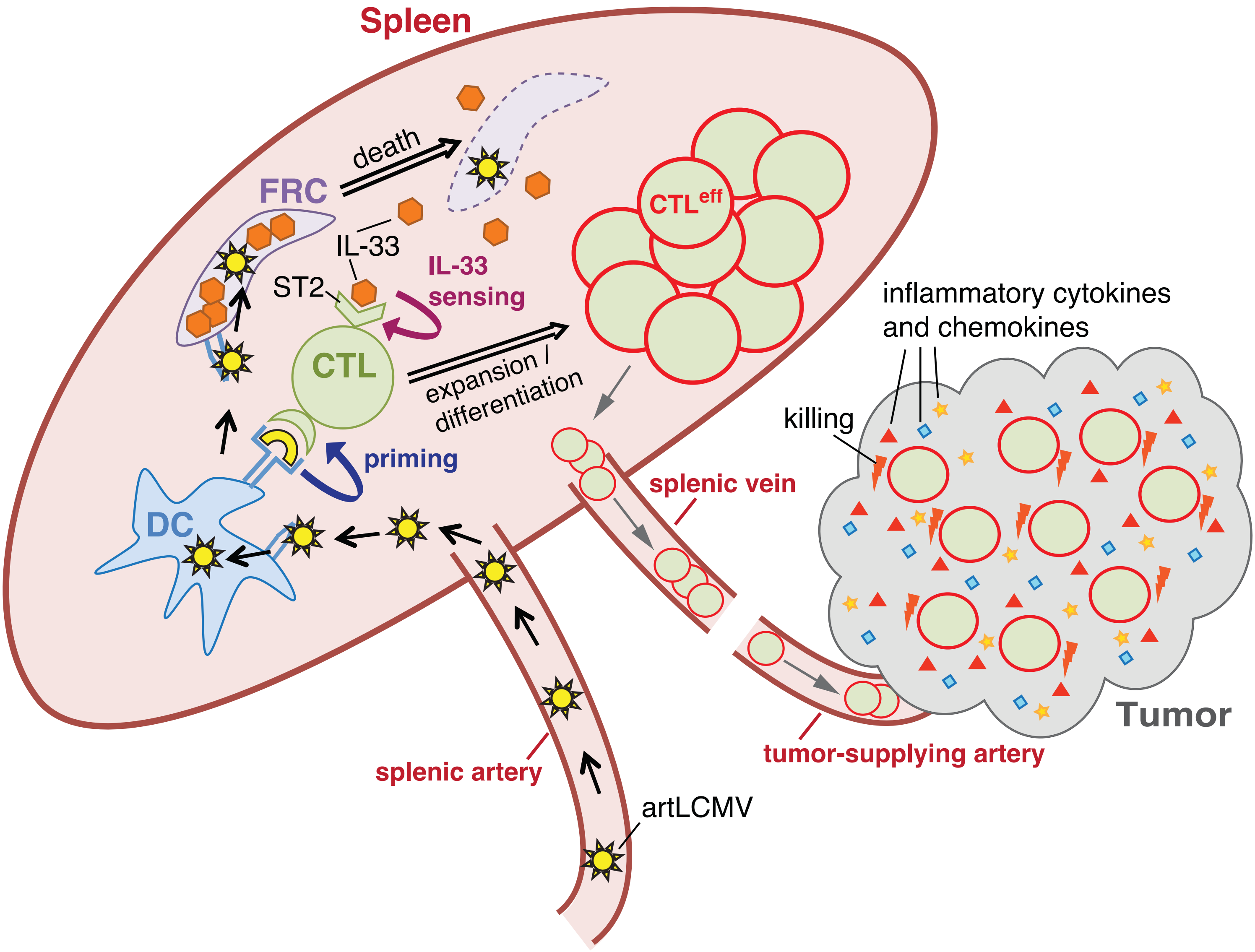

Supplementary Figure 6: Suggested mechanism of action of artLCMV tumor immunotherapy

Upon intravenous artLCMV administration, splenic DCs are a primary cellular target. Synthesis of artLCMV-encoded TAAs in DCs, alongside with virus-induced DC activation, results in potent $CD8^+$ T cell priming. Replication and secondary spread of replication-competent artLCMV inside the spleen leads to infection of stromal cells such as fibroblastic reticular cells (FRCs) in the T zone, which constitutively express IL-33. The death of these artLCMV-infected FRCs, presumably as a consequence of CTL attack[2], causes the release of bioactive IL-33. DC-primed TAA-specific CTLs express ST2 and sense IL-33, which potentiates their expansion, survival and effector differentiation[3]. High numbers of fully differentiated TAA-specific CTL^{eff} migrate through the bloodstream and home into the tumor, where they kill tumor cells and cause an inflammatory activation of the tumor microenvironment.

Supplementary References

1. Oboki K, *et al.* IL-33 is a crucial amplifier of innate rather than acquired immunity. *Proc Natl Acad Sci U S A* **107**, 18581-18586 (2010).

2. Scandella E, *et al.* Restoration of lymphoid organ integrity through the interaction of lymphoid tissue-inducer cells with stroma of the T cell zone. *Nat Immunol* **9**, 667-675 (2008).

3. Bonilla WV, *et al.* The alarmin interleukin-33 drives protective antiviral CD8(+) T cell responses. *Science* **335**, 984-989 (2012).
